# Left Bundle Branch Area Pacing in Older Patients: A New Opportunity?

**DOI:** 10.3390/life16030490

**Published:** 2026-03-17

**Authors:** Michele Alfieri, Lorenzo Pimpini, Filippo Pirani, Daniele Caraceni, Giulia Matacchione, Federico Guerra, Michela Casella, Roberto Antonicelli

**Affiliations:** 1 Cardiology and Cardiac Intensive Care Unit, The Department of Clinical, Special and Dental Sciences, IRCCS, INRCA, 60127 Ancona, Italy; 2 The Department of Biomedical Sciences and Public Health, Polytechnic University of Marche, 60131 Ancona, Italy; 3 Arrhythmology and Cardiology Clinic, Marche Regional Hospital, 60126 Ancona, Italy; 4 Clinic of Laboratory and Precision Medicine, IRCCS INRCA, 60127 Ancona, Italy; 5 Department of Clinical, Special and Dental Sciences, Polytechnic University of Marche, 60131 Ancona, Italy

**Keywords:** left bundle branch area pacing, LBBAP, CRT, pacing, physiological pacing, resynchronization therapy, heart failure, older people, frailty

## Abstract

**Background**: Resynchronization therapy has become a cornerstone in patients with heart failure (HF). Recent advancements in this field have led to the development of the so-called “left bundle branch area pacing” (LBBAP), a form of pacing where a single ventricular catheter directly addresses the left bundle for a more physiological stimulation. The current literature provides encouraging evidence regarding this topic, but there is still limited data for the older population, particularly those aged ≥75 years. This review aims to clarify how LBBAP has been explored in this cohort and if its application could be safe and effective even in the most advanced stages of life. **Methods**: A search of articles from PubMed was conducted. Patients were considered older if above 75 years of age. Data regarding Italian statistics were obtained from national registries. **Results**: The current literature supports the safety and effectiveness of LBBAP in older patients across different indications, with outcomes comparable to those reported in younger patients and a suggested cost-effectiveness. Conversely, data regarding patients affected by cardiac amyloidosis are still inconclusive. **Conclusions**: LBBAP represents a valuable resource for patients of all ages, but frailty is a major issue in the older population that needs to be addressed. The potential integration of this technology with defibrillator capabilities will enable an even more extensive application in the near future.

## 1. Introduction

**Figure 1:**
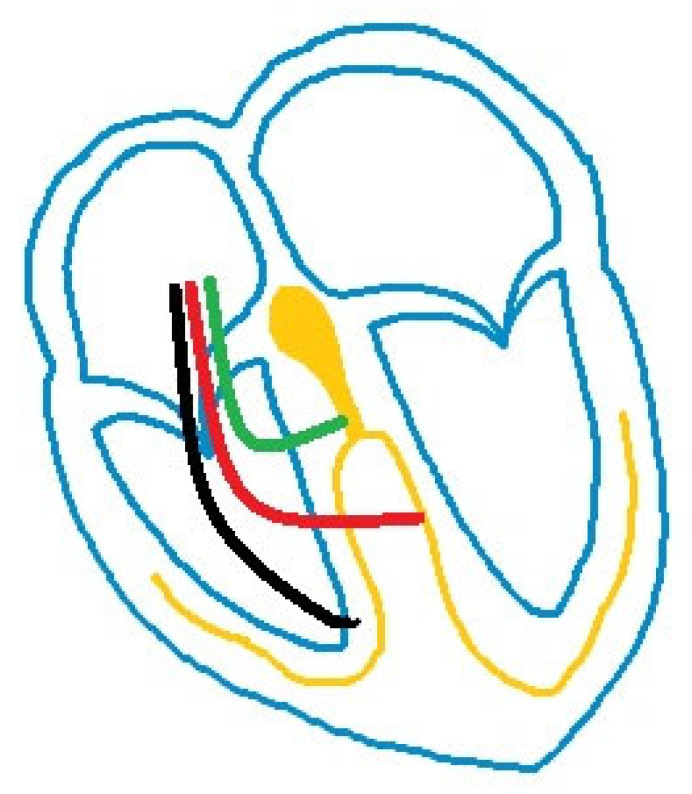
Graphic representation of different types of stimulation: in black RVP, in green HBP, in red LBBAP.

Heart failure (HF) is an increasing concern in Western countries, with a prevalence higher than 10% in patients above 70 years of age [1]. In recent years, advancements in pharmacological and device therapies have led to a significant improvement in survival, hospitalizations and quality of life. In approximately 30% of patients, an additional pathogenetic role is mediated by a prolonged QRS duration, which, in turn, induces asynchronous ventricular activation and remodeling [2,3]. The same pathogenetic role is responsible for pacing-induced cardiomyopathy (PICM). In this condition, pacing from the right ventricle (RVP) induces a slower electrical propagation, thereby widening the QRS and, in turn, changing the onset and order of mechanical activation with a progressive deterioration of ventricular structure and function [4]. In such cases, an established treatment is cardiac resynchronization therapy (CRT), which can be integrated with a defibrillator catheter (CRT-D) or performed without one (CRT-P). This device requires the insertion of a catheter into the coronary sinus, in addition to a classical bicameral pacemaker, in order to narrow the QRS by stimulating both ventricles (biventricular pacing; BVP) and improving ventricular mechanics. Many trials have already confirmed the efficacy of CRT in different settings, and the older population seems to be no exception: patients above 75 show significant improvements in quality of life, ventricular function and prognosis in a fashion comparable to younger populations [5,6,7,8], although patients with higher frailty scores show less encouraging responses [9]. In patients in whom CRT is not feasible, an immediate alternative is His-bundle pacing (HBP). In HBP there is a single catheter directly stimulating the His bundle, thereby improving intraventricular conduction; the main limitations of this therapy are its low effectiveness in patients with infra-nodal blocks and the high thresholds frequently requiring the insertion of a backup catheter, which, in turn, increases procedural risks and device-related costs [10]. In order to overcome the limitations posed by both CRT and HBP, a new kind of resynchronization therapy, called “left bundle branch area pacing” (LBBAP) has made its entrance in recent years (Figure 1). The innovation of this device lies in the selective capture of the conduction system after His-bundle division, namely the left bundle (left bundle branch pacing; LBBP), one of its ramifications (left fascicular pacing; LFP) or the nearby myocardium (left ventricular septal pacing; LVSP).

The current data on LBBAP seem to be promising, but little is known regarding its applicability and effectiveness in patients over 75 years. In this review, we aim to summarize the current evidence on left bundle branch area pacing in patients aged 75 years and older, focusing on feasibility, safety, and clinical effectiveness. In addition, we discuss the potential advantages of this technique over conventional resynchronization strategies and highlight existing gaps in the literature, particularly in relation to frailty and specific disease subsets in the older population.

## 2. Materials and Methods

The literature search was conducted in PubMed using the following Boolean strings: left bundle branch area pacing and elderly, LBBAP and elderly, physiological pacing and elderly, left bundle branch area pacing and octogenarians, left bundle branch pacing and octogenarians, left bundle branch area pacing and right ventricular pacing and older patients, safety and left bundle branch pacing and advance aged patients, safety and left bundle branch pacing and advanced age patients, left bundle branch area pacing and right ventricular pacing, left bundle branch area pacing prevents pacing-induced cardiomyopathy, left bundle branch area pacing vs. right ventricular pacing, left bundle branch area pacing and energetics. The articles included were published from January 2020 to December 2025. The filters applied were “Clinical Trial”, “Meta-Analysis”, “Observational Study”, and “Randomized Controlled Trial”. Articles were included if the mean age of the population was ≥75 years and selected based on their appropriateness and coherence with the topic. Studies with a mixed distribution but mean age above 75 were deemed suitable. No limits regarding the number of patients enrolled in the studies were adopted. The selection process was carried out by two different authors to reduce selection biases. Of the approximately 190 articles screened, 13 were included, while 36 were excluded for having a mean age <75 years, and 141 were excluded for lack of relevance to the topic. Data on Italian epidemiology and implants were drawn from the Italian National Healthcare Institute.

## 3. Definition of “Older Patients”

The commonly used cutoff to define a person as “older” has historically been set at 65 years of age. This convention does not accurately reflect the actual burden of comorbidities or frailty and should therefore be interpreted with caution, particularly in an era of markedly increased life expectancy. As early as 2006, Orimo suggested a shift from 65 to 75 years by taking into account functional and structural observations [11]. Interestingly, a meta-analysis regarding one of the most used scores for predicting frailty in hospitalized patients, the Hospital Frailty Risk Score (HFRS), identified how, in patients with cardiovascular and cerebrovascular diseases, the average age of the high frailty risk group ranged from 75.1 to 84.6 [12]. On the other hand, it is important to bear in mind that frailty is a clinical syndrome encompassing a series of conditions beyond age per se. Indeed, the World Health Organization (WHO), despite considering “older” individuals above 60, introduced the concept of “healthy aging”, which takes into consideration the preservation of functional abilities even in advanced stages of life. In conclusion, caution is warranted when applying strict age cutoffs, and additional variables, including the geographical context, should be taken into account. In this review, we adopted a threshold of ≥ 75 years to define the older population, as it may better reflect a more contemporary characterization of aging.

## 4. Feasibility of LBBAP in Older Populations

As with a pacemaker, in LBBAP there is a single catheter, which is placed and screwed inside the interventricular septum (IVS), reaching the left ventricular sub-endocardium; the mechanism underlying its efficacy lies in the selective capture of the left bundle, one of its branches or the nearby myocardium [13]. The correct placement is usually checked through fluoroscopy and QRS morphology by continuous pacing; during the advancement of the wire, the QRS complex should progressively shorten with a positive polarity in V1 [14]. Avoiding the placement of an additional lead into the coronary sinus is particularly fascinating in older and more frail patients, as it may reduce the need for contrast media and shorten the procedure, thereby reducing the risk of complications such as phrenic capture or catheter displacement. A recent study from Jiang and colleagues compared the safety and applicability of LBBAP in patients both older than and younger than 80 years of age and found no differences between electrical parameters or procedural complications [15]. Another study conducted from 2017 to 2019 showed that, despite patients over 80 years old having more comorbidities and carrying a worse NYHA class, their feasibility and clinical effectiveness were comparable to their younger counterparts [16]. A further study analyzing LBBAP in conjunction with atrio-ventricular node ablation (ablate and pace) in older patients (79.2 ± 4.2 years old) with atrial fibrillation (AF) showed no complications or lead stability issues [17] (Table 1). In conclusion, there is currently no substantial evidence against the safety and applicability of such technology in advanced age, but these findings need to be addressed carefully considering the limited number of patients included.

## 5. Effectiveness

The effectiveness of LBBAP has already been compared to other resynchronization strategies [18]. In the HOT-CRT trial, both HBP and LBBAP showed superiority in systolic improvement compared to BVP [19]. Another observational study including over 1700 patients concluded that LBBAP carries superior outcomes in patients with HF and an indication to resynchronization therapy [20]. A meta-analysis including 17 studies showed lower mortality rates, fewer HF hospitalizations and better LVEF improvements in patients treated with LBBAP compared to BVP [21]. Unfortunately, those studies did not focus on older patients, the population that most frequently requires a form of resynchronization strategy in the context of HF.

### 5.1. LBBAP in Older Patients with HF

Data from different studies seem to be oriented in the same direction. A single-center prospective study by Grieco et al. showed that in patients ≥ 75 years, LBBAP was successful in improving left ventricular structure (namely end-diastolic and end-systolic diameter) and ejection fraction (LVEF) without differences in complications, pacing thresholds, sensing and lead impedance [22]. Comparable results emerged from an observational study in 11 octogenarians, where an increase in LVEF from 47.6 ± 11.2% to 58.4 ± 3.7% was observed after 10 months of follow-up [23]. An additional retrospective study by Korkmaz showed, in patients ≥ 80 years, LVEF improvement, QRS duration and complication rates similar to those in younger individuals, despite longer fluoroscopy times [24] (Table 2).

More controversial is the role of LBBAP in the context of the most prevalent cardiomyopathy in older patients: cardiac amyloidosis (CA). In a single case series of CA individuals (from 84 to 92 years of age), satisfactory results were reported in 2 out of 3 patients in terms of echocardiographic and electrical parameters after 18 months [25], while another study enrolling 23 patients with a mean age of 78.6 years old reported a 95% implant success rate with an effective reduction in QRS width, but without significant changes in LVEF or NT-proBNP during a mean follow-up of 7.7 months [26]. In addition to CA, another limitation of this device in patients with systolic dysfunction is the need for a defibrillator. Indeed, LBBAP does not currently integrate a defibrillator into its system. A proposed solution might be the integration of LBBAP and CRT-D in the so-called “left bundle branch pacing optimized cardiac resynchronization therapy” (LOT-CRT). This solution is relatively unexplored in the older population and consists of the implantation of a CRT-D in conjunction with an LBBAP catheter. The strategies that can be adopted when integrating the two systems involve the insertion of three ventricular catheters: the LBBAP catheter, the coronary sinus catheter and the shock catheter; conversely, when there is no need for a shock catheter, an IVS catheter can be used as a right ventricular lead [27]. A case report in a patient aged 81 years who had an insufficient response to HBP showed positive outcomes with a narrower QRS and a significant improvement in NYHA class and LVEF [28]. Another case report described the application of a similar approach in a 76-year-old [29]; however, in this article, a different method was used to integrate the two pacing devices: the insertion of a shock catheter directly into the IVS. Notably, valuable clinical and instrumental outcomes were reported, including an increase in LVEF from 21 to 63%.

### 5.2. Paradigm Shift: LBBAP or Pacemaker?

**Figure 2:**
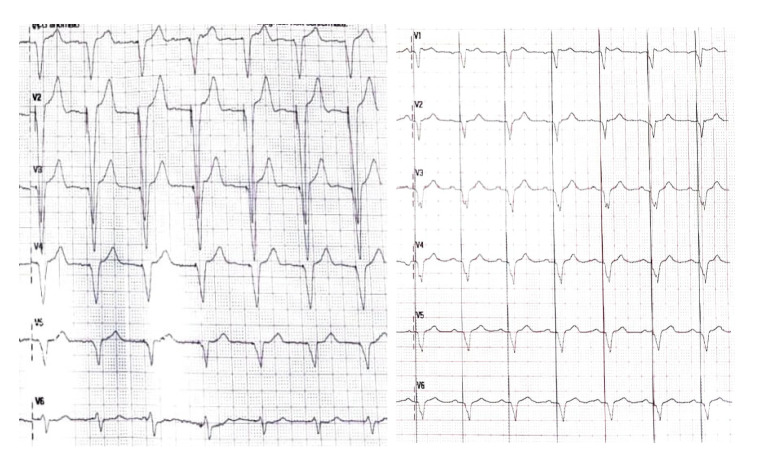
Different pacing modalities: on the left RVP, on the right LBBAP.

This technology can be applied to conventional pacemaker indications (Figure 2). The more physiological stimulation induced by LBBAP may prevent the development of PICM, which usually occurs in patients with a pacemaker carrying a high ventricular pacing burden; early data from observational registries seem to confirm this statement [30]. Notably, a retrospective study analyzing cardiac energetics by using magnetic resonance showed that LBBAP induces a leftward shift in the cardiac pressure–volume loop compared to patients with RVP, thus reflecting an improved stroke work and a reduction in myocardial oxygen consumption. Remarkably, in this paper, patients treated with LBBAP ranged from 75 to 83 years old [31]. In another recent prospective observational study by Wang and colleagues, in older patients (80.8 ± 4 years old) with symptomatic bradycardia, LBBAP preserved LVEF and reduced a composite outcome of all-cause mortality, HF hospitalization and upgrade to BVP compared to classical right ventricular stimulation [32]. In this study the difference between the groups was significant at a pacing burden of >20%. A study by Sharma and colleagues showed a reduction in all-cause mortality, HF hospitalizations and the need for biventricular pacing in patients with a pacing burden >20% compared to RVP, although the population also included younger individuals (mean age 75.13 ± 12 years) [30]. The same outcome was observed in a subsequent prospective study enrolling patients with a high pacing burden due to atrio-ventricular block (mean age 77.2 ± 11.0 years) [33]. Similar results were also reported in an additional single-center study exploring patients affected by conduction defects post-TAVI implantation (mean age of 77.9 ± 7.3 years), with a significant reduction in NT-proBNP levels and QRS duration while preserving systolic function [34]. A further study analyzing 151 patients (76.1 ± 11.8 years old) in need of ventricular pacing without an indication for CRT concluded that LBBAP improves LVEF after a mean follow-up of 23 months [35]; interestingly, age did not influence the increase in LVEF at the multivariate analysis. More convincing results come from the recent MELOS RELOADED trial, enrolling 3382 patients with atrio-ventricular block and a mean age of 76.4 ± 12.3 years, which showed a reduction in all-cause mortality with LBBAP compared to RVP after 4 years of follow-up [36]. Interestingly, in this study, older patients were uniformly distributed between the groups, but age was a predictor of increased mortality (Table 3).

Furthermore, a small amount of evidence suggests that physiological stimulation may carry a further benefit: a study including older individuals reported a higher incidence of AF both in patients treated with right ventricular septal stimulation (mean age 76.9 ± 7.0 years) and right ventricular apical stimulation (mean age 79.1 ± 8.3 years) compared to His pacing [37]; however, data regarding elderly patients treated with LBBAP are still scarce on this matter.

## 6. Discussion

This is, to our knowledge, the first review analyzing the role of LBBAP in older patients. In this context, LBBAP seems to represent a valid alternative to both right ventricular pacing and CRT, with a high success rate. As for all new technologies, practice matters: Wang et al. demonstrated that the learning curve reaches a stable state after approximately 150 procedures [38], thus highlighting the need for a proper operator experience.

### 6.1. Type of Stimulation

Another factor that needs to be taken into consideration is the type of capture. There is still uncertainty regarding the best type of bundle stimulation: capturing the left bundle before bifurcation, one of the fascicles or the septal myocardium may present different outcomes. Cheng and colleagues demonstrated the non-inferiority of LVSP compared to LBBP or LFP in terms of cardiac mechanics, but the number of patients enrolled was relatively low, and the group treated with LVSP had a mean age of 74.9 ± 7.7 years [39]. Furthermore, according to recent data from the MELOS RELOADED trial, LVSP is associated with worse outcomes than LBBP, but still better than RVP after a follow-up of 4 years [36]. Another study analyzing the catheter position through CT scan analysis (mean age 63 years) revealed better outcomes if the lead tip was closer to the LV endocardium or the His bundle [40]. Further data are needed to clarify this aspect, but, to date, there are no clear indications regarding the best site.

### 6.2. LBBAP and HF

One of the observations raised in this review is that LBBAP seems to reduce, in patients with a pacing indication, the incidence of PICM. The older population is particularly at risk of such occurrence considering its significant rate of conduction disturbances and the frequently high pacing burden. There are also some conditions necessitating a high pacing burden by design, such as in patients treated with atrio-ventricular node ablation; in these cases, LBBAP may find a more widespread application by optimizing stroke work and preserving systolic dysfunction with the use of a single catheter. Furthermore, considering that a net clinical benefit becomes relevant with a pacing burden > 20% [32], its applications might be extended in the near future. A further interesting possibility is the potential of this technology to reduce the burden and progression of AF compared to RVP [41]. It is possible that the more physiological mechanics induced by capturing the left bundle would be responsible for a disharmonic ventricular relaxation on top of systolic dysfunction; interestingly, the increase in left ventricular filling pressures and systolic dysfunction are directly related to atrial pump failure, thereby inducing more frequent atrial remodeling and, in turn, AF [42]. This is particularly relevant in older patients, considering the association of AF with hospitalizations, vascular dementia and mortality [43]; however, whether LBBAP could reduce the incidence of AF in older patients is yet to be determined. An important issue to be highlighted in the older population is the high prevalence of cardiac amyloidosis after 75 years of age. Indeed, despite the feasibility reported in the current literature, robust evidence regarding the effectiveness of LBBAP is still lacking, and the potential for therapeutic futility remains a concern in this subset of patients. As already pointed out, the small amount of evidence in this field is still controversial [25,26]; it is possible that the favorable results obtained by Mehta and colleagues in their case series are reflective of the longer follow-up period (18 vs. 7.7 months), but the low sample sizes of both studies prevent us from making generalizations. We speculate that the possible lack of improvement in amyloidotic hearts may come from a structural, rather than a functional, alteration with a hampered responsiveness to electrical and mechanical stimuli secondary to amyloid infiltration, but this remains only a hypothesis. To date, there are no clear indications regarding the extensive application of LBBAP in patients affected by CA, and further studies with a higher number of patients are needed to solve this particular matter.

### 6.3. Further Considerations

In addition to its clinical effects, physiological pacing may carry further advantages. A single study [44] compared the cost-effectiveness ratio (CER, obtained as cost/echocardiographic response rate) between LBBAP and BVP. Remarkably, LBBAP performed better in terms of echographic improvement, hospitalization costs and length of hospitalization. CER was 346,8 for BVP compared to 112,7 for LBBAP, thus suggesting a 3-fold reduction in costs for the same result. Taking into account the number of patients affected by HF in Italy (approximately 600,000 [45]) and the consequent number of CRT devices implanted (almost 9000 in Italy in 2023 [46]), there might be a potential impact on healthcare management costs. Notably, such data do not take specifically into account the older population, and, most importantly, CER might not be applicable to all settings and healthcare systems. Furthermore, a clear projection of national savings cannot be made based on these data. Until new evidence becomes available, the extensibility of such observations remains speculative and needs confirmation in older and larger cohorts.

### 6.4. Limitations

Despite these positive aspects, there are also downsides. One of the main drawbacks of LBBAP in HF with a reduced ejection fraction is the indication for a defibrillator; in these cases LOT-CRT represents a possible solution, but robust data on the older population are still scarce and the possible complications stemming from both the insertion of a coronary sinus catheter and an IVS catheter (on top of the increase in costs) make it less attractive. On the other hand, it is always important to check whether a patient may benefit from a defibrillator by taking into account comorbidities and quality of life; in this case, frailty is a major concern, and the risk of performing an inappropriate procedure might be tangible without a proper pre-assessment. In addition, LBBAP is not free from risks: the MELOS study reported an overall complication burden similar to CRT (11.7%), including minor events. Such complications include those specific to the trans-septal route of the device (8.3%), such as acute coronary syndromes (ACS; 0.48%), acute (3.67%) or delayed (0.08%) septal perforation, coronary artery fistulas (0.08%), chest pain (0.98%) and ST elevation (0.24%), and non-specific complications such as pneumothorax (0.55%), infections (0.24%) and vein thrombosis (0.08%) [47]. Notably, in this article, age was associated with lead implantation failure at the univariate analysis, but the population, despite including older patients, had a mean age of 73.9 ±11.8 years. A possible explanation for this outcome might be the different composition and strength of an “older” IVS, which could be more prone to trans-septal complications, but this statement does not find fertile ground in the current literature: a CT-scan study showed that, among both healthy and pathological hearts, age does not influence septal densitometry per se, but, notably, the mean age of the population enrolled was lower than 75 years [48]. In a recent study analyzing patients with septal injury after LBBAP implantation, after 26 septal perforations, 3 septal venous channel perforations and 3 coronary artery fistulas, all patients stayed asymptomatic without septal kinetics anomalies or adverse outcomes [49], but, once again, this paper did not specifically address the older population (70 ± 17 years old). The MELOS study reports a similar outcome, with most complications managed conservatively and with the need for coronary intervention in just one case [48]. In order to prevent septal perforation, a recent study provided a possible strategy in patients with a mean age of 81.4 years. According to this paper, while screwing the catheter, frequently checking not only QRS morphology but also the current of injury is of pivotal importance since its sudden drop may be reflective of septal perforation [50]. Further useful parameters are the drop in unipolar impedance, worsening of ventricular capture, loss of LBB or of the fascicular potential and contrast passage into the left ventricle [14].

### 6.5. LBBAP and Frailty

An aspect that needs to be addressed in the older population is whether the patient would really benefit from a resynchronization strategy. Even in CRT patients, frailty has been shown to be an independent predictor of all-cause mortality and HF-related hospitalizations [9,51]. A study by Kathi and colleagues including over 2900 patients analyzed the outcomes of resynchronization therapy in frail patients using the Rockwood method. According to their results, frailty is more frequently related to an ischemic etiology, a worse NYHA class, worse right ventricular function and an increased incidence of the final outcome (all-cause mortality). Notably, in this study, the mean age of patients in the high frailty group was 70 years, highlighting the marginal role of chronological age per se and the central importance of an appropriate frailty assessment [52]. The same results were obtained from another paper using the modified frailty index, which reported a higher mortality and a failure to respond to CRT with a score ≥ 3 [53]. In another study published in 2021, frailty was an independent predictor of death in patients treated with CRT irrespective of the presence of a defibrillator [54]. A meta-analysis including 15 studies involving patients treated with CRT confirmed such results and concluded that frail patients have a significant increase in mortality and HF decompensation compared to their counterparts [55]. Notably, in the population of patients over 80 years of age, the current literature does not suggest different outcomes compared to patients aged 75–80, even if based on a restricted number of studies. It is in fact crucial to understand the importance of an experienced team in managing highly complex patients with multiple comorbidities and that “chronological age” does not need to limit the best possible treatments. An intricate evaluation of what has been called “biological age” is the proper way to address most of the problems in the cohort of older individuals. Several scores have been developed to analyze health and frailty in older patients; a proper tool needs to take into account specific issues like mental health, activity and the level of dependency in everyday life. In our clinical practice, we currently use the Clinical Frailty Scale (CFS), which, even in its simplified version [56], is able not only to predict mortality but also to assess mobility, cognition, mental health and global function [57]. Moreover, its performance has been assessed in several populations and diseases encompassing different cardiological conditions. In summary, we generally do not recommend the application of a specific score, but we strongly suggest performing a frailty evaluation including mobility and independence in everyday activities. There is no precise guideline regarding the choice of a type of stimulation; the strength of the indication to a resynchronization strategy, the potential need for a defibrillator, the expected pacing burden, the underlying cardiac disease and overall clinical status must guide the electrophysiologist to pick a specific device over the others. In general, we suggest reducing the length of the procedure and the number of catheters alongside as frailty scores increase. In our opinion, in patients with limited functional capacity and no meaningful life expectancy, the decision to pursue a resynchronization strategy should be individualized, taking into account not only implantation costs but also device-related risks, life expectancy, operator expertise, and, most importantly, the expected clinical benefit.

## 7. Conclusions

The scarcity of dedicated studies on the matter prevents us from making precise recommendations, but based on the available data, we can draw some initial conclusions. First, LBBAP implantation seems to be a safe procedure even in patients ≥ 75 years old. Its effectiveness has also been shown in some clinical settings, mostly in patients with HF and in the prevention of PICM in individuals with an expected high pacing burden. On the other hand, the benefit in patients with CA is yet to be determined considering the scarcity and controversy of current evidence, while data regarding cost-effectiveness and AF incidence are still inconclusive. In addition, the absence of a shock catheter may be prohibitive in some patients. Complications are not rare, but their occurrence seems to be comparable to other resynchronization strategies. Despite being considered a major issue, chronological age does not seem to limit the feasibility or effectiveness of LBBAP, but a proper frailty assessment is of pivotal importance in order to avoid futility and adverse events. The current literature appears to be optimistic toward a more extensive application of this technology, but, in older patients, a multiparametric analysis of global frailty should be integrated to assess the potential benefit of this specific therapy.

## 8. Future Directions and Limitations

This review has several limitations that should be acknowledged. First, the generalizability of the conclusions is constrained by the limited number of available studies, which are predominantly derived from single-center experiences. Moreover, although efforts were made to ensure transparency and objectivity in data interpretation, the fact that this review was largely conducted within a single center means that the presence of confirmation bias cannot be entirely excluded. In addition, the cost-effectiveness analysis is taken from a single study and, therefore, its applicability on a large scale is yet to be demonstrated and, at this time, remains a hypothesis. Despite these limitations, LBBAP appears to offer several potential advantages in terms of applicability, procedural safety, effectiveness, and cost. As such, this technology may find broader adoption among patients of different ages, both with and without HF. Furthermore, the integration of defibrillator capabilities and remote monitoring may allow for a more timely management of arrhythmic complications and dynamic therapy optimization, potentially overcoming some of the limitations associated with conventional CRT and HBP while preserving comparable clinical benefits.

## Figures and Tables

**Table 1:** Articles on safety of LBBAP in older patients.

Article	Mean Age	Outcome	Year
Jiang et al. [15]	84	No difference in safety or complications compared to patients with mean age of 66	2022
Ren et al. [16]	84	No difference in safety or complications compared to patients with mean age of 72	2021
Rijks et al. [17]	79	No complications or lead stability issues after AV-node ablation	2023

**Table 2:** Articles on LBBAP in older patients with HF.

Article	Mean Age	Outcome	Year
Grieco et al. [22]	81	No difference in safety or LVEF increase compared to patients with mean age of 66	2023
Ponnusamy et al. [23]	82	Significant increase in LVEF from 47.6 to 58.4	2021
Korkmaz et al. [24]	82	Similar increase in LVEF compared to patients with a mean age of 62	2025

**Table 3:** Articles on LBBAP in place of RVP in older patients.

Article	Mean Age	Outcome	Year
Sharma et al. [30]	75	LBBAP improves outcomes compared to RVP in pacing burden > 20%	2022
Inoue et al. [31]	79	LBBAP-treated patients show better pressure–volume loop parameters	2025
Wang et al. [32]	81	LBBAP shows preserved LVEF and improved clinical outcomes compared to RVP	2024
Okubo et al. [33]	77	LBBAP improves outcomes compared to RVP in patients with a high pacing burden	2025
Vela-Martìn et al. [34]	78	LBBAP preserved LVEF and shortened QRS after TAVI	2025
Bednarek et al. [35]	76	LBBAP prevented PICM in patients with pacing burden > 40%	2023
Jastrzębski et al. [36]	76	LBBAP reduced mortality compared to RVP	2025

## Data Availability

Data not available due to privacy restrictions.
